# 2023 National Clinical Database Annual Report by the Japan Surgical Society

**DOI:** 10.1007/s00595-024-02980-1

**Published:** 2025-01-17

**Authors:** Takehito Yamamoto, Arata Takahashi, Tomoharu Yoshizumi, Soichiro Ishihara, Masafumi Inomata, Shigeru Imoto, Hidetoshi Eguchi, Tomoki Ebata, Masayuki Otsuka, Hiroomi Okuyama, Yoshihiro Kakeji, Tatsuya Kato, Takashi Kamei, Yoshikatsu Saiki, Aya Saito, Hideyuki Shimizu, Yoshiharu Soga, Tatsuro Tajiri, Hiroko Nogi, Etsuro Hatano, Hisato Hara, Yuko Bitoh, Tsunekazu Mizushima, Kenji Minatoya, Shigeru Miyagawa, Hideko Yamauchi, Ichiro Yoshino, Hideo Baba, Hisahiro Matsubara, Kiyoshi Hasegawa, Akinobu Taketomi

**Affiliations:** 1 Japan Surgical Society, Tokyo, Japan; 2 Department of Surgery, Graduate School of Medicine, Kyoto University, Kyoto, Japan; 3 Department of Gastroenterological Surgery and Oncology, Medical Research Institute Kitano Hospital, Osaka, Japan; 4 Department of Healthcare Quality Assessment, Graduate School of Medicine, The University of Tokyo, Tokyo, Japan; 5 Department of Surgery and Science, Graduate School of Medical Sciences, Kyushu University, Fukuoka, Japan; 6 Department of Surgical Oncology, Graduate School of Medicine, The University of Tokyo, Tokyo, Japan; 7 Department of Gastroenterological and Pediatric Surgery, Faculty of Medicine, Oita University, Oita, Japan; 8 Department of Breast Surgery, Kyorin University School of Medicine, Tokyo, Japan; 9 Department of Gastroenterological Surgery, Osaka University Graduate School of Medicine, Osaka, Japan; 10 Division of Surgical Oncology, Nagoya University Graduate School of Medicine, Aichi, Japan; 11 Department of General Surgery, Graduate School of Medicine, Chiba University, Chiba, Japan; 12 Department of Pediatric Surgery, Osaka University Graduate School of Medicine, Osaka, Japan; 13 Division of Gastrointestinal Surgery, Department of Surgery, Kobe University Graduate School of Medicine, Hyogo, Japan; 14 Department of Thoracic Surgery, Hokkaido University Graduate School of Medicine, Hokkaido, Japan; 15 Department of Surgery, Tohoku University Graduate School of Medicine, Miyagi, Japan; 16 Division of Cardiovascular Surgery, Tohoku University Graduate School of Medicine, Miyagi, Japan; 17 Department of Surgery, Yokohama City University Graduate School of Medicine, Kanagawa, Japan; 18 Department of Cardiovascular Surgery, Keio University School of Medicine, Tokyo, Japan; 19 Department of Cardiovascular Surgery, Graduate School of Medical and Dental Sciences, Kagoshima University, Kagoshima, Japan; 20 Department of Pediatric Surgery, Graduate School of Medical Sciences, Kyushu University, Fukuoka, Japan; 21 Department of Breast and Endocrine Surgery, Jikei University School of Medicine, Tokyo, Japan; 22 Division of Hepato-Biliary-Pancreatic Surgery and Transplantation, Department of Surgery, Kyoto University Graduate School of Medicine, Kyoto, Japan; 23 Institute of Medicine, Breast and Endocrine Surgery, University of Tsukuba, Ibaraki, Japan; 24 Division of Pediatric Surgery, Department of Surgery, Kobe University Graduate School of Medicine, Hyogo, Japan; 25 Department of Colorectal Surgery, Dokkyo Medical University, Tochigi, Japan; 26 Department of Cardiovascular Surgery, Graduate School of Medicine, Kyoto University, Kyoto, Japan; 27 Department of Cardiovascular Surgery, Osaka University Graduate School of Medicine, Osaka, Japan; 28 University of Hawai’i Cancer Center, Honolulu, USA; 29 International University of Health and Welfare Narita Hospital, Chiba, Japan; 30 The Chemo-Sero-Therapeutic Research Institute, Kumamoto, Japan; 31 Department of Frontier Surgery, Graduate School of Medicine, Chiba University, Chiba, Japan; 32 Hepato-Biliary-Pancreatic Surgery Division, Department of Surgery, Graduate School of Medicine, The University of Tokyo, Tokyo, Japan; 33 Department of Gastroenterological Surgery I, Hokkaido University Graduate School of Medicine, Hokkaido, Japan

**Keywords:** Japan Surgical Society, JSS, National Clinical Database, NCD

The Japan Surgical Society (JSS), founded in 1889, represents the largest organization of surgeons in Japan and encompasses diverse fields in surgery. Since 2011, the JSS has conducted annual surveys of the number of procedures performed in each surgical category throughout Japan, based on the National Clinical Database (NCD) and has presented its official webpage every year as a reference for Japanese medical professionals [1]. The NCD is a nationwide web-based data entry system that began in 2010 with 3374 participating institutions and 4916 clinical departments, recording approximately 1.17 million cases in its first year. As of April 2024, the number of participating institutions had increased to 5679, with 14,920 clinical departments [2]. The NCD captures over 95% of the relevant surgical procedures performed in Japan and its input data have been utilized in the management of surgical outcomes across various medical specialty certification systems.

The present report summarizes, for the first time in *Surgery Today*, the results of the 2023 JSS survey of the NCD. The data were extracted within a secure system with no external connections. When interpreting the number of surgeries, the following points should be noted: First, in the NCD, up to eight procedures can be selected for a single case; therefore, the simple sum of the number of procedures does not equate to the total number of surgical cases; second, the NCD includes some internal medicine-related treatments, making it difficult to capture the total number of such procedures accurately; third, certain procedures may be counted in other fields as well; and fourth, procedures with fewer than 20 cases were excluded from publication.

Procedure names were classified in detail based on NCD definitions, and many surgeries for the same anatomical site were divided into multiple distinct names; for example, colectomy was categorized as total colectomy, subtotal colectomy, partial colectomy, hemicolectomy, extended colectomy for a malignant tumor, and simple colectomy for a malignant tumor. Some of these surgeries were divided further into laparoscopic and open procedures.

## Survey abstract

All data were obtained from the NCD. During 2023, approximately 2.63 million cases were registered, bringing the cumulative total to around 28.48 million [2].

The surgical procedures included in the present report were classified into seven categories covered by JSS:

(I) Gastroenterological and abdominal surgery
(II) Breast surgery
(III) General thoracic surgery
(IV) Cardiovascular surgery
(V) Peripheral vascular surgery
(VI) Surgery of the head and neck/body surface/endocrine system
(VII) Pediatric surgery.

Figure 1 lists the number of cases in each category. All tables in this report list surgical procedures arranged in descending order of the number of surgeries performed.

**Fig. 1:**
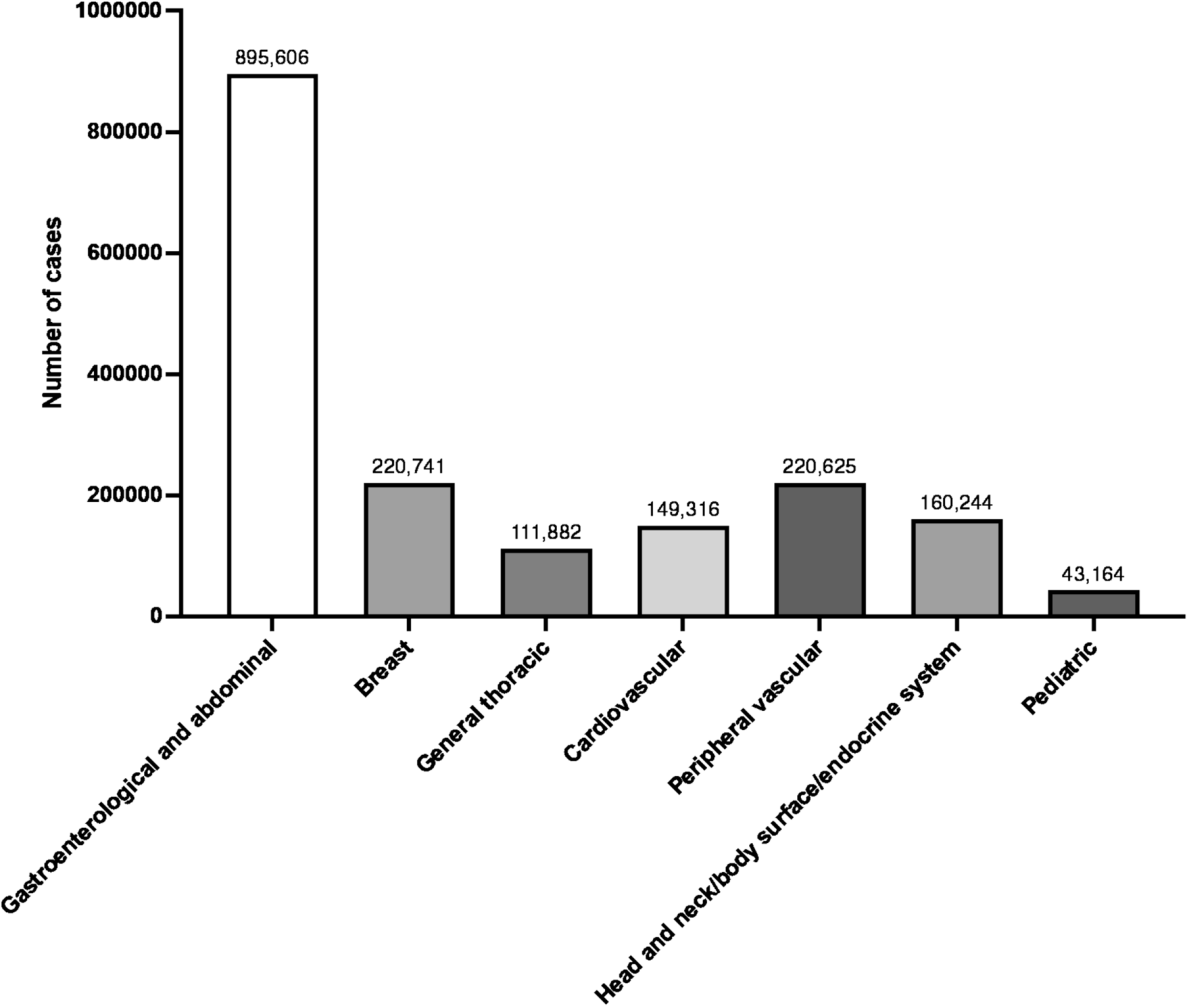
The number of cases in the seven surgical categories covered by the Japan Surgical Society

### (I) Gastroenterological and abdominal surgery

In 2023, there were 895,606 gastroenterological and abdominal surgical procedures registered in the NCD. The procedure with the highest number in this category was laparoscopic cholecystectomy (*n* = 118,825), followed by laparoscopic inguinal hernia surgery (*n* = 82,669), and open inguinal hernia surgery (*n* = 63,234).

Tables 1, 2, 3, 4, 5, 6, 7, 8, 9 and Fig. 2 show the number of surgeries in each subcategory (esophagus; stomach and duodenum; intestine, appendix and colon; rectum and anus; liver, bile duct and pancreas; spleen; abdominal cavity and peritoneum; transplantation; and other gastroenterological and abdominal surgery, respectively).

**Table 1:** Esophagus (total; 10,350 cases)

Procedure	Case
Thoracoscopic esophagectomy with gastrointestinal reconstruction (involving cervical, thoracic, and abdominal procedures, without vascular anastomosis) for a malignant tumor	4658
Esophageal hiatus hernia surgery (laparoscopic)	1184
Thoracoscopic esophagectomy with gastrointestinal reconstruction (involving thoracic and abdominal procedures, without vascular anastomosis) for a malignant tumor	576
Laparoscopic fundoplication	480
Thoracoscopic esophagectomy with gastrointestinal reconstruction (involving cervical, thoracic, and abdominal procedures, with vascular anastomosis) for a malignant tumor	343
Mediastinoscopic esophagectomy with gastrointestinal reconstruction (involving cervical, thoracic, and abdominal procedures, without vascular anastomosis) for a malignant tumor	283
Endoscopic esophagectomy for malignant tumor (resection only)	276
Esophageal hiatus hernia surgery (transabdominal)	247
Esophageal fistula construction	191
Secondary esophageal reconstruction	187
Open esophagectomy with gastrointestinal reconstruction (involving thoracic and abdominal procedures) for a malignant tumor	171
Open esophagectomy with gastrointestinal reconstruction (involving cervical, thoracic, and abdominal procedures, without vascular anastomosis) for a malignant tumor	151
Laparoscopic fundoplication	148
Esophagectomy (resection only) of the thoracic esophagus	146
Secondary gastrointestinal reconstruction following esophagectomy (with vascular anastomosis)	126
Esophagectomy with gastrointestinal reconstruction (involving abdominal procedures, including a transhiatal approach) for a malignant tumor	101
Esophageal reconstruction with bypass creation	90
Esophageal suture (perforation, injury) (thoracotomy)	86
Cervical esophagectomy with gastrointestinal reconstruction (involving cervical and abdominal procedures) for a malignant tumor	77
Mediastinoscopic esophagectomy with gastrointestinal reconstruction (involving thoracic and abdominal procedures, without vascular anastomosis) for a malignant tumor	69
Esophagectomy (resection only) of the thoracic esophagus	68
Esophageal perforation or injury repair (via laparotomy)	63
Achalasia surgery (laparoscopic)	59
Esophageal suture (perforation, injury) (cervical approach)	59
Open fundoplication	56
Thoracoscopic benign esophageal tumor removal	42
Esophageal resection and reconstruction for benign disease (involving cervical, thoracic, and abdominal procedures)	41
Gastric tube resection	40
Esophagectomy (resection only) of the abdominal esophagus	40
Esophageal resection (resection only) of the abdominal esophagus	39
Cervical esophagectomy with gastrointestinal reconstruction (involving cervical, thoracic, and abdominal procedures) for a malignant tumor	35
Esophagojejunostomy with jejunal pouch interposition	35
Surgery for a malignant tumor of the larynx and hypopharynx, with reconstruction involving cervical, thoracic, and abdominal procedures	31
Esophagectomy (resection only) of the cervical esophagus	30
Esophageal resection and reconstruction for benign disease (involving abdominal procedures)	28
Cervical periesophageal abscess drainage	24
Esophageal foreign body extraction	24
Esophageal diverticulum resection	24
Mediastinoscopic esophageal resection with gastrointestinal reconstruction (without thoracotomy)	22
Total	10,350

**Table 2:** Stomach and duodenum (total; 59,822 cases)

Procedure	Case
Laparoscopic resection for gastric malignancy	17,422
Extended resection for gastric malignancy	7594
Laparoscopic total gastrectomy for gastric malignancy	3300
Extended total gastrectomy without pedicled bowel transplantation	3183
Gastrojejunostomy	2913
Gastrointestinal anastomosis	2790
Omental filling or covering procedure for gastric or duodenal ulcer perforation	2305
Laparoscopic local gastrectomy	2166
Gastric fistula construction	1820
Proximal gastrectomy for malignancy	1667
Laparoscopic-assisted gastrostomy (including percutaneous endoscopic, percutaneous, and open approaches)	1548
Omental filling or covering procedure	1337
Laparoscopic endoscopic cooperative local gastrectomy	1294
Simple resection for gastric malignancy	1210
Duodenal suture (perforation, injury) (laparoscopic)	934
Gastric suture (perforation, injury) (laparotomy)	853
Laparoscopic sleeve gastrectomy	851
Extended proximal gastrectomy for gastric malignancy	827
Open local gastrectomy	826
Simple total gastrectomy for gastric malignancy	799
Completion total gastrectomy	684
Open duodenal repair (for perforation, rupture, or injury)	628
Gastrectomy for non-malignant disease	522
Gastric suture (perforation, injury) (laparoscopic)	508
Duodenojejunostomy	290
Extended total gastrectomy with pedicled bowel transplantation	258
Surgery for hypertrophic pyloric stenosis	251
Open gastrostomy closure	133
Open pyloroplasty	115
Laparoscopic extended total gastrectomy with a jejunal pouch	99
Completion gastrectomy	95
Pylorus-preserving gastrectomy	95
Gastric foreign body extraction	74
Open duodenal polypectomy	71
Total gastrectomy for benign disease	58
Laparoscopic surgery for hypertrophic pyloric stenosis	56
Laparoscopic surgery for gastric volvulus	44
Gastric reduction surgery	43
Laparoscopic sleeve gastrectomy with bypass	41
Surgery for gastric volvulus	41
Duodenal diverticulum resection	39
Proximal gastrectomy for benign disease	38
Total	59,822

**Table 3:** Intestine, appendix, and colon (total; 251,395 cases)

Procedure	Case
Laparoscopic colectomy for a malignant tumor	48,885
Laparoscopic simple appendectomy	37,312
Stoma creation	33,421
Extended colectomy for a malignant tumor	16,103
Open small bowel resection	15,913
Laparoscopic complex appendectomy	14,465
Open surgery for intestinal adhesions	12,958
Stoma closure with enterectomy	11,732
Open partial colectomy	8972
Laparoscopic surgery for intestinal adhesions	7617
Laparoscopic partial colectomy	6033
Open appendectomy	5031
Laparoscopic small bowel resection	4599
Enterostomy creation	4309
Intestinal anastomosis	3441
Hemicolectomy	3410
Simple colectomy for malignant tumor	3012
Laparoscopic colectomy	2681
Stoma closure without enterectomy	2052
Stoma closure after Hartmann's procedure	1242
Simple small bowel resection for malignant tumor	812
Subtotal colectomy	669
Laparoscopic small bowel resection for malignant tumor	646
Gastrointestinal perforation closure	466
Total colectomy	432
Colostomy creation	429
Enterostomy closure with enterectomy	417
Extended small bowel resection for malignant tumor	393
Total colectomy with ileoanal anastomosis	324
Enterotomy for foreign body extraction	252
Surgery for intestinal malrotation	245
Meckel's diverticulectomy	237
Open stoma revision surgery	236
Disinvagination (invasive)	216
Small bowel seromuscular suture	207
Non-open stoma revision	199
Laparoscopic total colectomy	197
Enterostomy closure without enterectomy	172
Colon tumor resection	168
Small bowel tumor resection	149
Laparoscopic enterostomy and appendicostomy creation	139
Simple colonic suture	116
Small bowel diverticulectomy	102
Laparoscopic disinvagination	99
Colostomy closure with colectomy	98
Surgery for congenital intestinal atresia with bowel resection	91
Colonic seromuscular suture	87
Total proctocolectomy with ileal pouch-anal anastomosis	80
Laparoscopic surgery for intestinal malrotation	73
Enterotomy and suture repair for intestinal stricture	72
Colostomy closure without colectomy	72
Laparoscopic Hirschsprung disease surgery	57
Complex mesenteric injury surgery with enterectomy	56
Simple mesenteric injury surgery with enterectomy	49
Surgery for intestinal duplication	35
Surgery for congenital intestinal atresia without bowel resection	35
Enterotomy for biopsy	31
Open colonic polypectomy	25
Umbilical enteric fistula surgery with bowel resection	24
Total	251,395

**Table 4:** Rectum and anus (total; 157,776 cases)

Procedure	Case
Radical hemorrhoid surgery	37,770
Internal hemorrhoid surgery (four-step injection)	18,679
Radical surgery for complex fistula-in-ano	12,088
Laparoscopic low anterior resection	9476
Incision and drainage of perianal abscess	9097
Radical surgery for simple fistula-in-ano	8281
Low rectal surgery for malignant tumor (extensive resection)	8059
Laparoscopic high anterior resection	5912
High rectal surgery for malignant tumor (extensive resection)	5143
Hemorrhoid surgery (ligation)	4386
Laparoscopic abdominoperineal resection	3783
Anal polyp resection	3431
Radical surgery for anal fissure or ulcer	3398
Rectal prolapse surgery (transanal approach)	2966
Rectal resection	2697
Hemorrhoid surgery (sclerotherapy)	2292
Anoplasty (rectal mucosal prolapse repair)	2218
Hemorrhoid surgery (thrombectomy)	2183
Laparoscopic rectal prolapse surgery	1919
Anoplasty (for anal stricture)	1521
Rectal surgery for malignant tumor (extended amputation)	1368
Anal dilation with internal sphincterotomy (invasive)	1356
Transanal rectal tumor resection (including polyps)	1248
Ultra-low anterior resection with transanal coloanal pouch anastomosis	1171
Incision and drainage of perirectal abscess	1065
Open abdominoperineal resection	725
Hemorrhoid surgery (PPH)	694
Anal condyloma acuminatum removal	670
Abdominoperineal rectal prolapse surgery (including bowel resection)	563
Benign anal tumor resection	495
Rectal prolapse surgery (rectopexy)	435
Rectal surgery for malignant tumor (simple resection)	416
Total pelvic exenteration	409
Pilonidal cyst or sinus surgery	301
Transanal anal stricture dilation	247
Anal sphincteroplasty (tissue replacement)	180
Full-thickness rectal biopsy	179
Anal sphincteroplasty (scar excision or repair)	177
Anal surgery for malignant tumor (simple resection)	137
Transanal intraluminal rectal tumor resection (including polyps)	131
Partial rectal resection	119
Hemorrhoid surgery (cauterization)	117
Simple rectal suture	78
Rectal stricture repair	45
Open rectal foreign body extraction	41
Rectal seromuscular suture	31
Rectal surgery for malignant tumor (extensive resection with sacral resection)	28
Transsphincteric rectal tumor resection (including polyps)	26
Anal surgery for malignant tumor (extended resection)	25
Total	157,776

*PPH* procedure for prolapse and hemorrhoids

**Table 5:** Liver, bile duct and pancreas (total; 186,128 cases)

Procedure	Case
Laparoscopic cholecystectomy	118,825
Open cholecystectomy	14,972
Pancreaticoduodenectomy with lymphadenectomy and nerve plexus dissection	8365
Laparoscopic partial hepatectomy	6440
Partial hepatectomy	5904
Laparoscopic distal pancreatectomy with splenectomy	2603
Hepatectomy (bi-sectionectomy)	2376
Distal pancreatectomy with lymphadenectomy and nerve plexus dissection	1747
Hepatectomy (one-sectionectomy, excluding left lateral sectionectomy)	1720
Distal pancreatectomy with splenectomy	1528
Pancreaticoduodenectomy with arterial or portal vein reconstruction	1443
Hepatic segmentectomy	1436
Laparoscopic hepatic segmentectomy	1173
Malignant gallbladder tumor surgery (with resection of gallbladder bed)	1091
Laparoscopic hepatic cyst fenestration	1090
Pancreaticoduodenectomy	1000
Choledochotomy with stone extraction	984
Choledochoenterostomy	830
Laparoscopic pancreaticoduodenectomy	719
Surgery for localized malignant gallbladder tumor	689
Laparoscopic hepatectomy (bi-sectionectomy)	672
Laparoscopic hepatectomy (one-sectionectomy, excluding left lateral sectionectomy)	643
Laparoscopic choledochotomy with stone extraction	626
Hepatic left lateral sectionectomy	614
Laparoscopic hepatic left lateral sectionectomy	608
Distal pancreatectomy with resection of adjacent organs	507
Liver biopsy (needle puncture)	498
Resection of bile duct malignancy with hepatectomy	473
Laparoscopic spleen-preserving distal pancreatectomy	473
Total pancreatectomy without vascular reconstruction	458
Vascular (or venous) grafting or bypass	420
Pancreatojejunostomy	367
Pancreaticoduodenectomy with resection of adjacent organs	328
Hilar cholangiocarcinoma resection without vascular reconstruction	298
Bile duct malignancy resection with lymphadenectomy	290
Gallbladder malignancy resection with hepatectomy (segment 4a + 5 or more)	236
Extrahepatic bile duct resection with cholecystectomy and bile duct reconstruction	235
Bile duct resection	224
External cholecystostomy	216
Pancreaticoduodenectomy with artery and portal vein reconstruction	196
Central pancreatectomy	189
Spleen-preserving distal pancreatomy	164
Pancreatic tumor resection	154
Hepatectomy (trisegmentectomy)	153
Resection of hilar cholangiocarcinoma with vascular reconstruction	136
Hepatorrhaphy	135
Malignant bile duct tumor surgery with hepatectomy and pancreaticoduodenectomy	131
Distal pancreatectomy with vascular reconstruction	124
External biliary fistula	114
Biliary tract reconstruction	109
Laparoscopic surgery for choledochal cyst	99
Bilioenteric anastomosis	99
Open hepatic cyst fenestration	92
Surgery for congenital biliary atresia	92
Caudate lobectomy	85
Cysticolithectomy	78
Laparoscopic transcholecystic extraction of bile duct stones	75
Robot-assisted surgery for choledochal cysts	75
Esophageal varices ligation	74
Malignant gallbladder tumor surgery with pancreaticoduodenectomy	62
Total pancreatectomy with arterial or portal vein reconstruction	61
Total pancreatectomy with simultaneous arterial and portal vein reconstruction	58
Duodenum-preserving pancreatic head resection	47
Choledochotomy	47
Jejunal interposition of bile duct	47
Ampullectomy	46
Hepatic cyst resection	41
Cholecystoenterostomy	41
Hepatectomy with vascular reconstruction	41
Hepatic injury ablation and coagulation hemostasis (including hemostatic agents)	39
Percutaneous drainage of liver abscess	35
Deceased donor pancreas procurement	32
Acute pancreatitis surgery	31
Pancreatic lithotomy (pancreatotomy)	27
Open transcystic choledocholithotomy	25
Malignant gallbladder tumor surgery (with hepatectomy and pancreaticoduodenectomy)	22
Internal biliary fistula closure	21
Total	186,218

**Table 6:** Spleen (total; 1744 cases)

Procedure	Case
Open splenectomy	1044
Laparoscopic splenectomy	647
Partial splenectomy	31
Splenectomy with esophageal varices	22
Total	1744

**Table 7:** Abdominal cavity and peritoneum (total; 213,774 cases)

Procedure	Case
Laparoscopic inguinal hernia surgery	82,669
Open inguinal hernia surgery	63,234
Acute diffuse peritonitis surgery with drainage of intraperitoneal abscess	13,781
Umbilical hernia surgery (diastasis recti)	9137
Exploratory laparoscopy for diagnosis	6835
Exploratory laparotomy for diagnosis and biopsy	6502
Open abdominal wall incisional hernia surgery	5460
Laparoscopic abdominal wall incisional hernia surgery	4537
Laparoscopic diffuse peritonitis surgery	3685
Open femoral hernia surgery	3271
Obturator hernia surgery	2135
Laparoscopic femoral hernia surgery	1849
Omental, mesenteric, and retroperitoneal tumor resection (without bowel resection)	1526
Endoscopic tumor biopsy	1107
Localized intraperitoneal abscess surgery (other types)	937
Localized intraperitoneal abscess surgery (appendiceal abscess)	913
Internal hernia surgery	788
Temporary vacuum packing abdominal closure	692
Omentectomy	559
Simple resection of retroperitoneal malignant tumor	494
Linea alba hernia surgery	451
Omental, mesenteric, and retroperitoneal tumor resection (with bowel resection)	407
Laparoscopic resection of urachus (cyst)	331
Laparoscopic retroperitoneal tumor resection	260
Extended resection of retroperitoneal malignant tumor	250
Surgery for mesenteric injury (suture repair only)	212
Localized intraperitoneal abscess surgery (Douglas pouch abscess)	156
Diaphragm suture repair (transabdominal, without patch reconstruction)	145
Surgery for abdominal wall fistula (communicating with the abdominal cavity)	145
Localized intraperitoneal abscess surgery (subphrenic abscess)	124
Thoracoscopic diaphragm plication	111
Diaphragm suture repair (transthoracic, without patch reconstruction)	98
Lumbar hernia surgery	97
Thoracoscopic diaphragm suture repair (without patch reconstruction)	92
Semilunar line hernia surgery	87
Decompressive laparotomy for abdominal compartment syndrome	75
Diaphragmatic hernia surgery	71
Multivisceral resection with peritonectomy	69
Abdominal wall reconstruction (including skin grafting)	62
Removal of perihepatic packing gauze	60
Perineal hernia surgery	50
Transabdominal diaphragm plication	50
Component separation technique	49
Laparoscopic diaphragm suture repair (without patch reconstruction)	48
Transthoracic diaphragm plication	44
Retroperitoneal abscess drainage	44
Combined thoracoabdominal diaphragm suture repair (without patch reconstruction)	25
Laparoscopic diaphragm plication	20
Total	213,744

**Table 8:** Transplantation (total; 2755 cases)

Procedure	Case
Renal transplantation	891
Laparoscopic donor nephrectomy	582
Living donor liver transplantation	354
Backtable preparation for living donor liver transplantation	197
Living donor right hemi-hepatectomy	158
Deceased donor liver transplantation	118
Liver procurement from a deceased donor	100
Living donor left hemi-hepatectomy	85
Living donor left lateral sectionectomy	80
Back table preparation for deceased donor liver transplantation	60
Kidney procurement from deceased donor	42
Deceased donor pancreas transplantation	36
Living donor left hemi-hepatectomy with segmentectomy 1 (caudate segmentectomy)	29
Living donor partial small bowel procurement	23
Total	2755

**Table 9:** Other gastroenterological and abdominal surgery (total; 11,802 cases)

Procedure	Case
Hartmann's procedure	6852
Open hemostasis surgery	1221
Abdominal paracentesis with filtration, concentration, and reinfusion	944
Percutaneous RFA for a malignant liver tumor	446
Stent placement in the small intestine, colon, or rectum	281
Damage control surgery for hemorrhagic shock in abdominal trauma	275
Disinvagination (Non-invasive)	247
Thoracotomy for hemostasis	184
Placement of an implantable catheter for continuous intraperitoneal infusion	169
Open RFA for a malignant liver tumor	134
Peroral endoscopic myotomy	115
Open MWA for a malignant liver tumor	111
Laparoscopic RFA for a malignant liver tumor	100
Liver hemostasis (gauze packing)	99
Thoracentesis with filtration, concentration, and reinfusion	97
Percutaneous MWA for a malignant liver tumor	94
Pylorus-preserving pancreaticoduodenectomy	59
Mechanical dilation for upper gastrointestinal anastomotic stricture	47
Gastric foreign body extraction with magnetic catheter	44
Esophageal banding	42
Laparoscopic high anorectal reconstruction for imperforate anus	42
Sacral nerve stimulator lead insertion	39
Umbilical fistula surgery without bowel resection	38
Gastric vessel ligation for acute gastric bleeding	38
Esophageal dilation with Tucker bougie	32
Esophageal foreign body extraction with a balloon	30
External pancreatic duct fistula creation	22
Total	11,802

*RFA* radiofrequency ablation, *MWA* microwave ablation

**Fig. 2:**
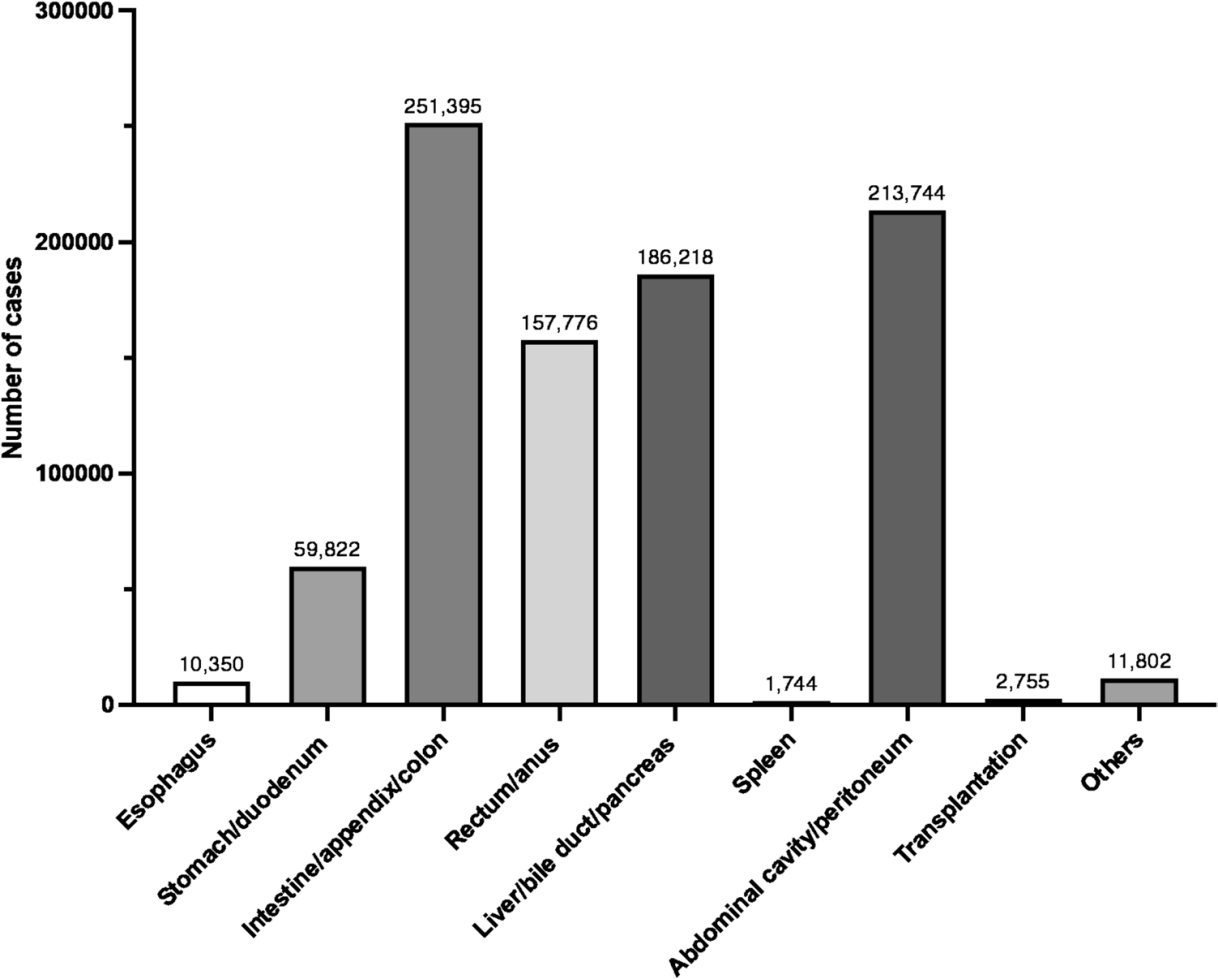
The number of cases in the category of gastroenterological and abdominal surgery

### (II) Breast surgery

In 2023, there were 220,741 breast surgery procedures registered in the NCD. Table 10 presents all the procedures in this category by volume. The procedure with the highest number in this category was sentinel lymph node biopsy for breast malignancy (*n* = 60,704), followed by mastectomy without axillary lymph node dissection (*n* = 39,498) and partial mastectomy without axillary lymph node dissection (*n* = 38,649), for breast malignancy. It should be noted that the total number of procedures does not correspond to the total number of cases, because multiple procedures, such as breast surgery and axillary surgery, may be registered for a single case.

**Table 10:** Breast surgery (total; 220,741 cases)

Procedure	Case
Sentinel lymph node biopsy for breast malignancy	60,704
Breast malignancy surgery: Mastectomy without axillary lymph node dissection	39,498
Breast malignancy surgery: Partial mastectomy without axillary lymph node dissection	38,649
Breast malignancy surgery: Mastectomy with axillary and infraclavicular lymph node dissection (without pectoral muscle resection)	18,287
Breast tissue biopsy by core needle biopsy	18,160
Image-guided vacuum-assisted breast biopsy	10,629
Breast tumor excision: Lesion < 5 cm (benign breast lesion)	5845
Lymph node excision: Lesion < 3 cm	5600
Breast malignancy surgery: Partial mastectomy with axillary lymph node dissection	5393
Axillary lymph node dissection	2954
Lymph node excision: Lesion ≥ 3 cm	2783
Breast tumor excision: Lesion ≥ 5 cm (benign breast lesion)	2615
Nipple-sparing mastectomy	1664
Immediate breast reconstruction after mastectomy (using implant)	1500
Skin-sparing mastectomy	1291
Breast malignancy surgery: Mastectomy with axillary and infraclavicular lymph node dissection (with pectoral muscle resection)	819
Mastectomy for benign breast lesion	720
Breast abscess drainage	668
Immediate breast reconstruction after mastectomy (using autologous tissue)	662
Delayed breast reconstruction (using implant)	556
Ductal lobular segmentectomy	483
Lymph node biopsy by core needle biopsy	403
Breast tissue biopsy by incisional biopsy (Incisional biopsy of breast tissue)	374
Supraclavicular and infraclavicular lymph node dissection	126
Excision of breast foreign body	108
Radical surgery for subareolar abscess	62
Nipple reconstruction (for inverted nipple)	62
Nipple reconstruction (for reconstructed breast)	41
Breast malignancy surgery: Extended mastectomy with internal mammary, supraclavicular, and infraclavicular lymph node dissection	32
Delayed breast reconstruction (using autologous tissue)	30
Parasternal lymph node dissection	23
Total	220,741

### (III) General thoracic surgery

In 2023, 111,882 general thoracic surgical procedures were registered in the NCD. The procedure with the highest number in this category was thoracoscopic lobectomy with lymph node dissection for a malignant lung tumor (*n* = 25,495), followed by thoracoscopic wedge resection for a malignant lung tumor (single site) (*n* = 14,686), and thoracoscopic bullectomy (single site) (*n* = 10,651). Tables 11, 12, 13, 14, 15 and Fig. 3 show the number of surgeries in each subcategory (trachea, bronchi, and lung; chest wall, pleura, and diaphragm; mediastinum; transplantation; and other respiratory surgery, respectively).

**Table 11:** Trachea, bronchi, and lung (total; 84,657 cases)

Procedure	Case
Thoracoscopic lobectomy with lymph node dissection for a malignant lung tumor	25,495
Thoracoscopic wedge resection for a malignant lung tumor (single site)	14,686
Thoracoscopic bullectomy (single site)	10,651
Thoracoscopic segmentectomy with lymph node dissection for a malignant lung tumor	8160
Thoracoscopic wedge resection (single site)	4460
Lobectomy with lymph node dissection for a malignant lung tumor (thoracotomy)	3726
Thoracoscopic segmentectomy without lymph node dissection for a malignant lung tumor	2703
Thoracoscopic bullectomy (two or more sites)	1944
Thoracoscopic pulmorrhaphy	1635
Thoracoscopic wedge resection for a malignant lung tumor (two or more sites)	1569
Thoracoscopic lobectomy without lymph node dissection for a malignant lung tumor	1101
Wedge resection for a malignant lung tumor	1056
Thoracoscopic wedge resection (two or more sites)	931
Thoracoscopic lobectomy	787
Thoracoscopic segmentectomy	770
Segmentectomy with lymph node dissection for a malignant lung tumor	690
Thoracoscopic lung biopsy	610
Wedge resection	578
Lobectomy	375
Segmentectomy without lymph node dissection for a malignant lung tumor	354
Lung resection with bronchoplasty for a malignant lung tumor	332
Pulmonary plication (thoracotomy)	288
Lung resection with chest wall for malignant lung tumor	266
Lobectomy without lymph node dissection for a malignant lung tumor (thoracotomy)	202
Closure of bronchopleural fistula	192
Thoracoscopic lung resection (more than one lobe)	175
Segmentectomy	130
Wedge resection with lymph node dissection for a malignant lung tumor	123
Lung resection with combined resection of other organs for a malignant lung tumor	101
Pneumonectomy with lymph node dissection for a malignant lung tumor (thoracotomy)	98
Thoracoscopic lung resection with combined resection of other organs for a malignant lung tumor	84
Thoracoscopic lung resection with chest wall resection for a malignant lung tumor	75
Thoracoscopic lung resection with bronchoplasty for a malignant lung tumor	61
Lung resection (combined resection of more than one lobe)	52
Pneumonectomy (complete removal of one lung)	43
Lung resection with combined resection of the chest wall, pericardium, or diaphragm for a malignant lung tumor	31
Lung resection with diaphragmatic resection for a malignant lung tumor	29
Lung resection with pericardial resection for a malignant lung tumor	26
Thoracoscopic pneumonectomy with lymph node dissection for a malignant lung tumor	23
Lung resection for extralobar pulmonary sequestration	23
Lung resection with pulmonary vascular reconstruction	22
Total	84,657

**Table 12:** Chest wall, pleura, and diaphragm (total; 8796 cases)

Procedure	Case
Thoracoscopic debridement for empyema	3040
Thoracoscopic decortication of empyema and pleural thickening	994
Evacuation of intrathoracic (intrapleural) hematoma	671
Evacuation of intrathoracic hematoma	461
Open window thoracostomy	335
Chest wall or sternum tumor resection for a malignant tumor (without chest wall reconstruction)	282
Thoracoscopic extirpation of a benign chest wall tumor	268
Partial pleurectomy	219
Pectus excavatum repair by sternal elevation	197
Decortication of empyema exceeding the range of one lobe	148
Diaphragm suture repair (transabdominal, without patch reconstruction)	145
Thoracoscopic resection of malignant pleural tumor	141
Space-filling of omentum flaps for an empyema cavity	141
Thoracoplasty for empyema (with rib resection)	135
Space-filling of pedicled muscle flaps for an empyema cavity	131
Thoracoscopic diaphragm plication	111
Decortication of empyema within the area of one pulmonary lobe	101
Resection of a malignant pleural tumor	100
Diaphragm suture repair (transthoracic, without patch reconstruction)	98
Chest wall/sternum resection for a malignant tumor (with chest wall reconstruction)	95
Thoracoscopic diaphragm suture repair (without patch reconstruction)	92
Thoracoscopic excision of a benign pleural tumor	89
Pleurectomy/Pulmonary decortication	83
Thoracoscopic thoracic duct ligation for chylothorax	78
Excision of a benign chest wall tumor (without thoracotomy)	66
Excision of a benign chest wall tumor (with thoracotomy)	58
Congenital diaphragmatic hernia repair (transabdominal, direct suture)	57
Laparoscopic diaphragm suture repair (without patch reconstruction)	48
Extended pleurectomy/decortication	44
Diaphragm plication (transthoracic)	44
Congenital diaphragmatic hernia repair (transabdominal, with synthetic patch)	43
Chest wall resection for a malignant tumor (chest wall reconstruction with vascular anastomosis)	42
Thoracoscopic surgery for congenital diaphragmatic hernia (direct suture)	39
Pulmonary decortication exceeding the area of one pulmonary lobe	39
Thoracoplasty for empyema (with pleural decortication)	33
Sternum resection	32
Combined thoracoabdominal diaphragm suture repair (without patch reconstruction)	25
Excision of a benign pleural tumor	24
Chest wall/sternum resection for a malignant tumor (reconstruction with musculocutaneous flap)	24
Surgery for chylothorax	23
Total	8796

**Table 13:** Mediastinum (total; 7030 cases)

Procedure	Case
Thoracoscopic resection of a mediastinal tumor	3251
Thoracoscopic resection of a malignant mediastinal tumor	1081
Thoracotomy resection of a mediastinal tumor	442
Thoracoscopic thymectomy	398
Malignant mediastinal tumor resection	308
Thoracoscopic extended thymectomy for myasthenia gravis	200
Malignant mediastinal tumor resection with combined resection of other organs	197
Thymectomy (thoracotomy)	172
Mediastinoscopic Mediastinal biopsy	158
Malignant mediastinal tumor resection with lung resection	141
Malignant mediastinal tumor resection with pericardial resection	136
Mediastinal abscess surgery	126
Extended thymectomy for myasthenia gravis (thoracotomy)	103
Thoracoscopic mediastinotomy	102
Trans-thoracic mediastinotomy	53
Malignant mediastinal tumor resection with superior vena cava resection	49
Non-thoracotomy resection of mediastinal tumor	47
Mediastinoscopic resection of mediastinal tumor	36
Cervical mediastinotomy	30
Total	7030

**Table 14:** Transplantation (total; 234 cases)

Procedure	Case
Single lung transplantation from a deceased donor	101
Bilateral lung transplantation from a deceased donor	77
Procurement of bilateral lungs from a deceased donor for transplantation	36
Procurement of a single lung from a deceased donor for transplantation	20
Total	234

**Table 15:** Other respiratory surgeries (total; 11,165 cases)

Procedure	Case
Exploratory thoracoscopy	2603
Thoracic drainage with continuous suction	1904
Thoracoscopic thoracic sympathectomy	1567
Exploratory thoracotomy	1295
Pericardiotomy	1031
Thoracoscopic mediastinal biopsy	598
Open reduction and fixation for rib fracture	374
Thoracoscopic pectus excavatum repair	311
Thoraco-peritoneal shunt valve placement	252
Evacuation of mediastinal hematoma	230
Thoracoscopic pericardiotomy	192
Thoracoscopic pleural adhesional ablation	129
Rib resection	113
Tracheostomy closure	84
Mediastinal lymphadenectomy	78
Tracheal stricture dilation	66
Open reduction and fixation for sternum fracture	56
Chest wall abscess incision and drainage	49
Extracorporeal membrane oxygenation (ECMO) initiation	42
Open mediastinal biopsy	33
Non-thoracotomy mediastinal biopsy	33
Intrathoracic foreign body removal	31
Surgery for tracheal stenosis	26
Lung biopsy by needle puncture	24
Extracorporeal membrane oxygenation (ECMO) removal	23
Tracheal foreign body removal under fiberscope guidance	21
Total	11,165

*ECMO* extracorporeal membrane oxygenation

**Fig. 3:**
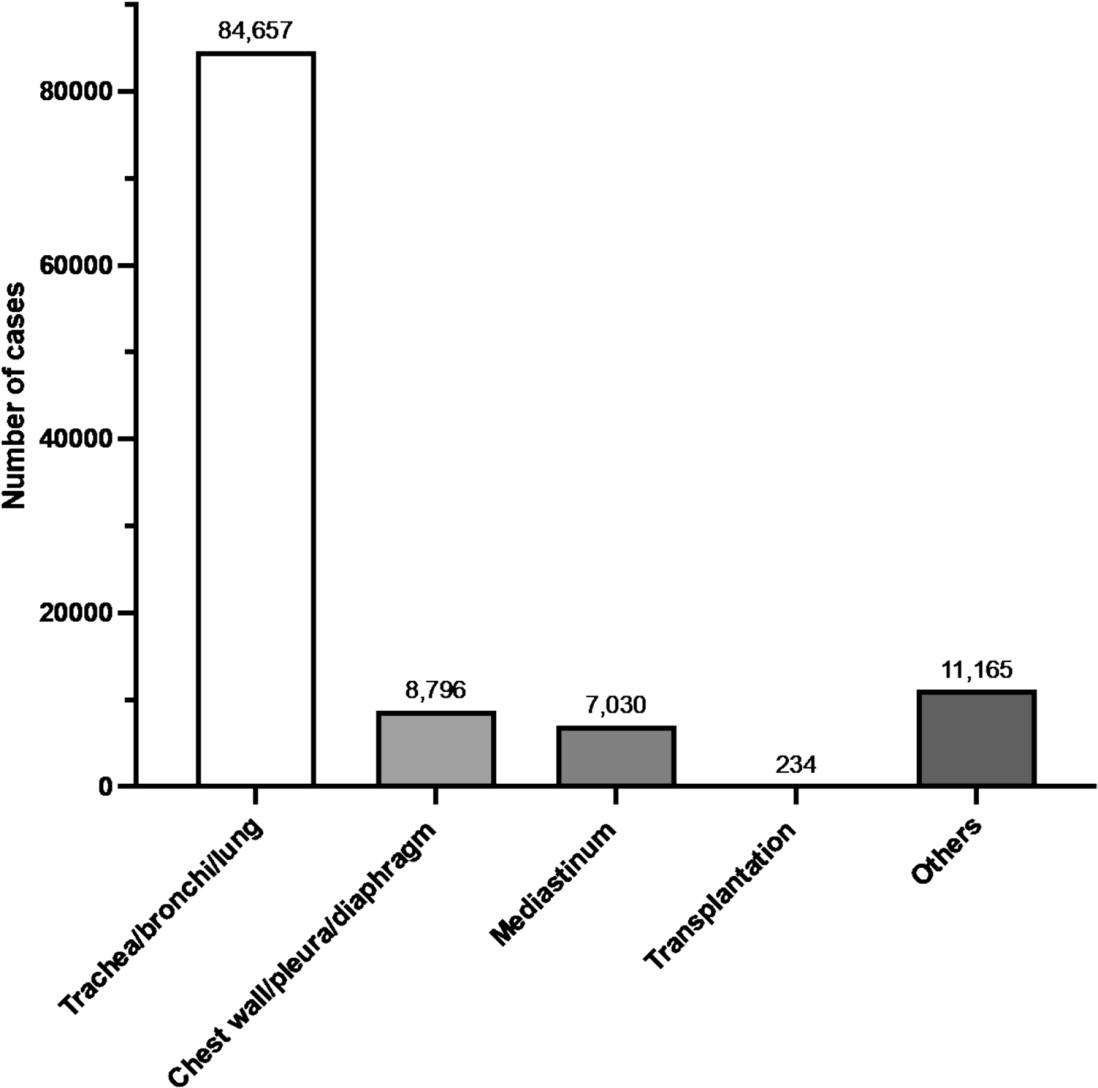
The number of cases in the category of general thoracic surgery

### (IV) Cardiovascular surgery

In 2023, 149,316 cardiovascular surgical procedures were registered in the NCD. The procedure with the highest number in this category was stent grafting of the abdominal aorta (*n* = 12,869), followed by transcatheter aortic valve replacement (TAVR) without thoracotomy (*n* = 10,696), and aortic valve replacement (AVR) (*n* = 9569). Tables 16, 17, 18, 19, 20, 21, 22, 23, 24, 25 and Fig. 4 show the number of surgeries in each subcategory (all heart diseases, congenital heart disease, valvular heart disease, ischemic heart disease, transplantation, other heart diseases, other cardiac surgeries, great vessels, great veins, and other great vessel surgeries, respectively).

**Table 16:** All heart diseases (total; 10,664 cases)

Procedure	Case
Thoracoscopic exploratory thoracotomy	2603
Median sternotomy	2365
Pacemaker implantation with transvenous leads	2261
Exploratory thoracotomy	1295
Pericardiotomy	1031
Pacemaker implantation with myocardial leads	293
Myocardial suture hemostasis for traumatic injury	207
Thoracoscopic pericardial fenestration	192
Pericardial closure	138
Pacemaker lead implantation	102
Biventricular pacemaker implantation	78
Bilateral anterolateral thoracotomy (clam shell thoracotomy)	55
Pacemaker lead replacement	44
Total	10,664

**Table 17:** Congenital heart disease (total; 8725 cases)

Procedure	Case
ASD closure (alone)	2030
VSD closure (alone)	1570
Pulmonary artery reconstruction (including pulmonary vein trunk and main pulmonary artery)	870
PDA closure (open surgery)	815
Pulmonary artery banding	500
Systemic-to-pulmonary artery shunt (Blalock-Taussig shunt)	469
Subvalvular aortic stenosis resection (including fibrous and muscular thickening)	297
Fontan procedure	257
Tetralogy of Fallot repair (with ventriculotomy)	252
Bilateral pulmonary artery banding	189
Aortic coarctation repair (isolated)	165
AVC (AVSD) repair, intermediate (transitional)	149
TAPVR repair (supracardiac and infracardiac types)	121
PAPVR repair	118
DORV repair (alone)	116
TGA repair (Rastelli procedure)	104
TGA repair (Jatene arterial switch operation)	86
Pulmonary artery de-banding	69
Partial AVSD repair (ASD patch closure only)	60
Coronary arteriovenous fistula ligation (via thoracotomy)	56
Aortic coarctation repair (with VSD closure)	54
Tricuspid atresia repair (Fontan procedure)	50
Valsalva sinus aneurysm repair (isolated)	50
Double-chambered right ventricle repair	47
Valsalva sinus aneurysm repair (with aortic insufficiency repair)	42
TAPVR repair (cardiac type)	37
Anomalous origin of coronary artery repair	36
Supravalvular aortic stenosis repair	34
Arterial switch operation (with VSD closure)	30
Interrupted aortic arch repair (alone)	28
Tricuspid valve surgery (for Ebstein anomaly)	24
Total	8725

*ASD* atrial septal defect, *VSD* ventricular septal defect, *PDA* patent ductus arteriosus, *AVC* atrioventricular canal, *AVSD* atrioventricular septal defect, *TAPVR* total anomalous pulmonary venous return, *PAPVR* partial anomalous pulmonary venous return, *DORV* double outlet right ventricle, *TGA* transposition of the great arteries

**Table 18:** Valvular heart disease (total; 41,371 cases)

Procedure	Case
Transcatheter aortic valve replacement (TAVR) without thoracotomy	10,696
Aortic valve replacement (AVR)	9569
Mitral valvuloplasty	5461
Mitral valve replacement (MVR)	2196
Tricuspid valvuloplasty	2080
Valvuloplasty (Mitral and tricuspid valves)	1393
Ascending aortic aneurysm repair with aortic root and valve replacement	1322
Ascending aortic aneurysm repair with aortic valve replacement or valvuloplasty	1266
Mitral valve replacement (MVR) and tricuspid valvuloplasty	1020
Ascending aortic and arch repair with aortic valve replacement or valvuloplasty	626
Mitral and tricuspid valvuloplasty	615
Aortic and mitral valve replacement	591
Valve-sparing aortic root replacement	468
Aortic valvuloplasty	411
Aortic valve replacement (AVR) and mitral valvuloplasty	381
Pulmonary valve replacement	371
Ascending aortic and arch repair with aortic root and valve replacement	369
Aortic and mitral valve replacement with tricuspid valvuloplasty	354
Aortic valve replacement with annular enlargement	351
Redo valve replacement (single valve)	347
Aortic valve replacement and tricuspid valvuloplasty	320
Transcatheter aortic valve replacement (TAVR) with thoracotomy	298
Aortic valve replacement and mitral and tricuspid valvuloplasty	281
Tricuspid valve replacement	170
Ascending aortic and arch repair with valve-sparing aortic root replacement	140
Mitral and tricuspid valve replacement	94
Triple valve replacement	44
Ross operation (aortic root replacement with pulmonary autograft)	42
Aortic and mitral valvuloplasty	36
Percutaneous transluminal mitral valvuloplasty	34
Redo valve replacement (two valves)	25
Total	41,371

**Table 19:** Ischemic heart disease (total; 17,762 cases)

Procedure	Case
CABG for two or more vessels	7180
Off-pump CABG for two or more vessels	6213
CABG for one vessel	2456
Off-pump CABG for one vessel	806
Ventricular septal rupture closure (alone)	239
Left ventricular free wall rupture repair (alone)	185
Surgical left ventricular restoration (alone)	168
Left ventricular free wall rupture repair	100
Ventricular septal rupture repair (alone)	68
Ventricular aneurysm reconstruction (including infarctectomy, alone)	58
Surgical ventricular restoration with CABG for two or more vessels	55
Coronary endarterectomy (1 site)	48
Ventricular septal rupture closure with CABG for one vessel	43
Surgical left ventricular restoration with CABG for one vessel	30
Ventricular aneurysm reconstruction with CABG for two or more vessels	26
Ventricular septal rupture closure with CABG for two or more vessels	24
Left ventricular free wall rupture repair with CABG for one vessel	23
Redo coronary artery bypass grafting	20
Intracardiac myxoma excision with CABG for one vessel	20
Total	17,762

*CABG* coronary artery bypass grafting

**Table 20:** Transplantation (total; 177 cases)

Procedure	Case
Allogeneic heart transplantation	116
Heart procurement for transplantation	61
Total	177

**Table 21:** Other heart diseases (total; 4554 cases)

Procedure	Case
Arrhythmia surgery (Maze procedure)	2533
Pulmonary vein isolation	1057
Intracardiac myxoma excision (alone)	592
Surgical treatment of constrictive pericarditis	174
Thrombectomy of pulmonary artery	97
Malignant pericardial tumor resection	46
Arrhythmia surgery (surgical division of accessory pathway with intraoperative electrophysiological study)	29
Arrhythmia surgery (surgery for ventricular tachycardia with intraoperative electrophysiological study)	26
Total	4554

**Table 22:** Other cardiac surgeries (total; 18,133)

Procedure	Case
Left atrial appendage closure	4048
Surgical removal of PCPS/ECMO	2780
Left atrial appendage excision	2537
Cardiopulmonary bypass setup	1371
Re-sternotomy for hemostasis	796
Surgical insertion of PCPS/ECMO	772
Open-chest cardiac massage	559
PCPS insertion	367
Left atrial plication (LAP)	344
Right ventricular outflow tract reconstruction	330
Intracardiac foreign body removal	254
Single-ventricle repair (bidirectional Glenn procedure)	241
ICD implantation	234
Mediastinal hematoma removal	230
Pulmonary artery debanding	228
Temporary epicardial pacing	228
Intra-atrial thrombus removal	205
Delayed sternal closure	193
Exploratory open-heart surgery	190
Percutaneous transluminal septal myocardial ablation (with additional procedures)	162
ICD replacement	160
Implantation of an implantable VAD	150
VAD placement	148
Open atrial septostomy	138
ICD (with biventricular pacing function) implantation	125
Norwood procedure for hypoplastic left heart syndrome	112
Conduit reoperation	96
Percutaneous transluminal septal myocardial ablation	86
Coronary artery aneurysm surgery	83
Retrograde coronary perfusion	81
VAD explantation	79
Implantable loop recorder placement	77
Right ventricle to pulmonary artery conduit placement	76
Transvenous lead extraction using laser sheath	69
Biventricular pacemaker replacement	60
Removal of implantable cardiac rhythm management device	51
Shunt ligation and takedown	50
Vascular ring surgery	50
Replacement of ICD with biventricular pacing function	50
Tricuspid atresia surgery (bidirectional Glenn procedure)	46
ECMO initiation	42
Unifocalization of the major aortopulmonary collateral arteries	36
Pericardial tumor resection	34
Adjustment of systemic-to-pulmonary artery shunt	33
ECMO removal	23
Division of double aortic arch	23
Ventricular assist device support (after 31 days)	23
Pulmonary atresia repair with Rastelli procedure	23
Aortopexy	20
Implantable loop recorder removal	20
Total	18,133

*PCPS* Percutaneous Cardiopulmonary Support, *ECMO* Extracorporeal Membrane Oxygenation, *ICD* implantable cardioverter-defibrillator, *VAD* ventricular assist device

**Table 23:** Great vessels (total; 20,751 cases)

Procedure	Case
Ascending aortic aneurysm replacement	3963
Abdominal aortic aneurysm replacement (with branch vessel reconstruction)	3087
Abdominal aortic aneurysm replacement (without branch vessel reconstruction)	3075
Total arch replacement	2821
Aortic aneurysm repair with frozen elephant trunk	2651
Combined surgery of ascending aortic and aortic arch repair	2071
Thoracoabdominal aortic aneurysm repair	764
Descending aortic aneurysm repair	592
Debranching (1–4 vessels) endovascular aneurysm repair	278
Graft replacement for ruptured abdominal aortic aneurysm	272
Vascular grafting or bypass for visceral artery	217
Graft replacement with renal artery clamp for abdominal aortic aneurysm	192
Abdominal aortic aneurysm replacement with renal artery clamping	186
Vascular grafting or bypass for aorta	172
Vascular grafting or bypass for intrathoracic artery	106
Stent grafting for aortic arch branches	84
Aneurysmorrhaphy	84
Aortic valvuloplasty or anastomosis	71
Vascular grafting or bypass for the iliac artery	65
Total	20,751

**Table 24:** Great veins (total; 358 cases)

Procedure	Case
Venous reconstruction of intra-abdominal vein	98
Venous anastomosis of intra-abdominal vein	93
Venous grafting or bypass surgery for intra-abdominal vein	89
Venous reconstruction of intrathoracic veins	50
Inferior vena cava injury repair with suture hemostasis	28
Total	358

**Table 25:** Other great vessel surgeries (total; 26,821 cases)

Procedure	Case
Stent grafting of the abdominal aorta	12,869
Surgical thrombectomy (artery, without thoracotomy or laparotomy)	6708
Stent grafting of the thoracic aorta	6197
Aortic cross-clamping (thoracotomy)	247
Inferior vena cava filter placement	184
Aortic cross-clamping with a balloon catheter	96
Thrombectomy (with laparotomy)	92
Pulmonary vein reconstruction	85
Pulmonary thromboendarterectomy	77
Stent grafting of the thoracoabdominal aorta	74
Open venous thrombectomy	66
Aortic coarctation surgery with Damus-Kaye-Stansel (DKS) anastomosis	49
Thoracic aortic wrapping	41
Thoracic aortic pseudoaneurysm repair	36
Total	26,821

**Fig. 4:**
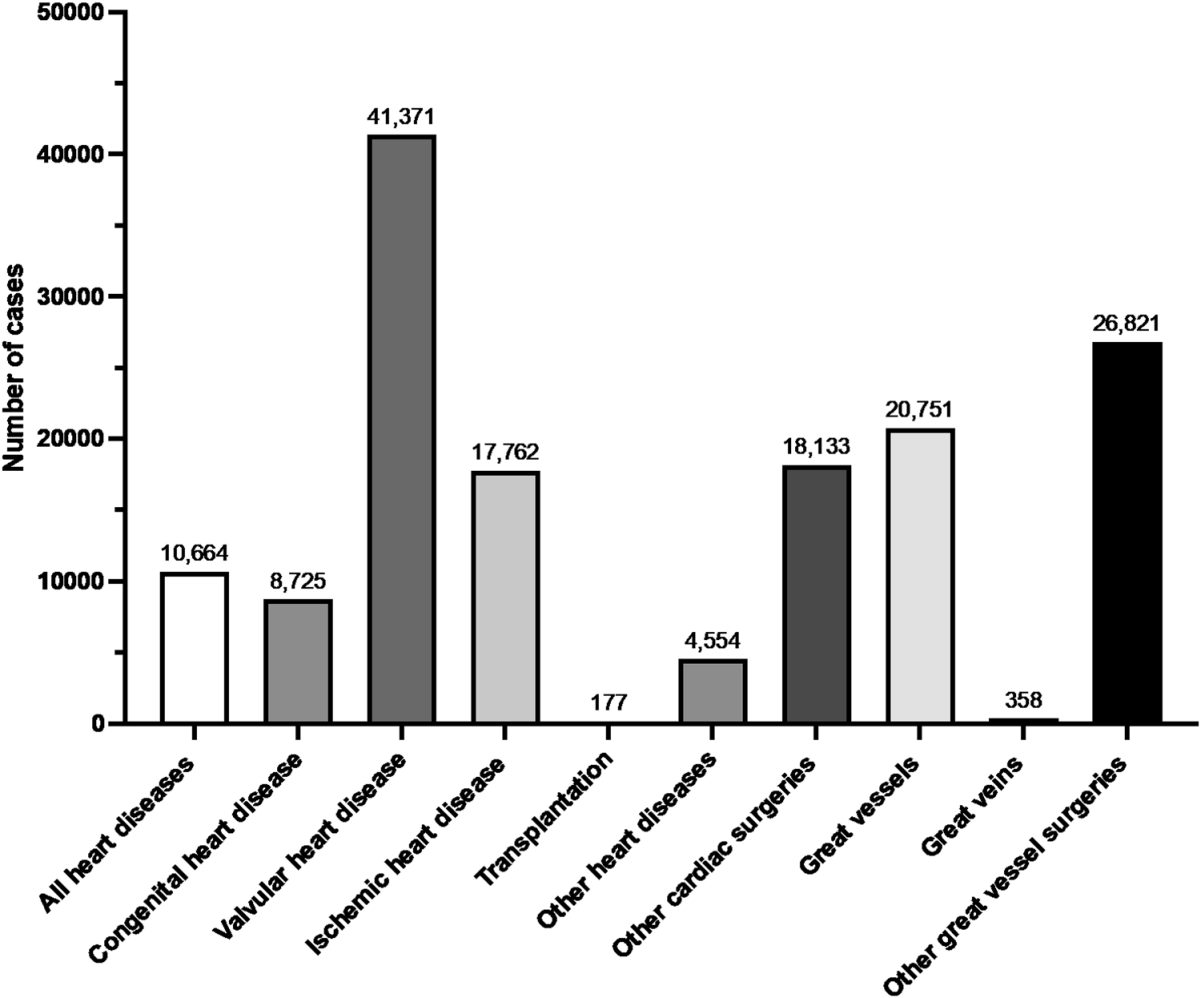
The number of cases in the category of cardiovascular surgery

### (V) Peripheral vascular surgery

In 2023, 220,625 peripheral vascular surgical procedures were registered in the NCD. The procedure with the highest number in this category was endovascular treatment for the extremity arteries (*n* = 54,798), followed by endovenous ablation of the lower extremity varicose veins (*n* = 30,153), and peripheral arteriovenous fistula creation (*n* = 22,180).

Tables 26, 27, 28, 29 and Fig. 5 show the number of surgeries in each subcategory (arteries, veins, other vascular systems, and other peripheral vascular surgery, respectively).

**Table 26:** Arteries (total; 105,468 cases)

Procedure	Case
Endovascular treatment for the extremity arteries	54,798
Stent grafting of the abdominal aorta	12,869
Surgical thrombectomy (without thoracotomy or laparotomy)	6708
Stent grafting of the thoracic aorta	6197
Thromboendarterectomy (other arteries)	3565
Abdominal aortic aneurysm replacement (with branch vessel reconstruction)	3087
Abdominal aortic aneurysm replacement (without branch vessel reconstruction)	3075
Arterial reconstruction or anastomosis (other arteries)	2867
Stent grafting of the iliac artery	2289
Arterial reconstruction or anastomosis for the femoropopliteal artery	1478
Vascular grafting or bypass for the femoral artery	1399
Vascular grafting or bypass for the tibial and foot arteries	1306
Vascular grafting or bypass for the below-knee popliteal artery	1151
Percutaneous angioplasty for the limb arteries (with a stent graft)	924
Debranching TEVAR with 1 or 2 bypass grafts	578
Peripheral artery suture hemostasis or anastomosis	499
Arterial reconstruction or anastomosis for the intra-abdominal arteries (excluding aorta)	390
Peripheral aneurysm resection (other types)	243
Peripheral aneurysm resection	218
Vascular grafting or bypass for the intra-abdominal arteries	217
Peripheral aneurysm resection (with anastomosis or grafting)	210
Percutaneous aspiration thrombectomy of the limb arteries	200
Percutaneous angioplasty for the renal artery	152
Arterial reconstruction or anastomosis for the finger arteries (hand or foot)	119
Fenestrated and branched TEVAR (with bridging-stent or stent grafts including in-situ fenestration)	103
Fenestrated and branched EVAR (with bridging-stent or -stent grafts, including iliac branch endoprosthesis [IBE])	88
Stent grafting for aortic arch branches	84
CHIMPS EVAR	81
Stent grafting for the visceral arteries	80
Stent grafting for the thoracoabdominal aorta	74
Vascular grafting or bypass for the iliac artery	65
Thromboendarterectomy (Aorta)	65
Percutaneous stent placement for the cervical and cerebral arteries	45
Carotid endarterectomy (CEA)	45
Renal artery revascularization	40
Fenestrated and branched thoracoabdominal EVAR (with bridging stent grafts)	39
Iliac artery suture hemostasis or anastomosis	35
CHIMPS TEVAR	35
Debranching TEVAR with 3 or more bypass grafts	29
Percutaneous balloon angioplasty for the mesenteric arteries	21
Total	105,468

*TEVAR* thoracic endovascular aortic repair, *EVAR* endovascular aneurysm repair, *CHIMPS* chimney, periscope, snorkel

**Table 27:** Veins (total; 39,011 cases)

Procedure	Case
Endovenous ablation of the lower extremity varicose veins	30,153
Excision of the lower extremity varicose veins	3802
Stripping of the lower extremity varicose veins	3089
Venous thrombectomy (non-laparotomy)	975
Venous grafting or bypass (other veins)	420
Percutaneous aspiration thrombectomy of extremity	295
Peripheral venous suture hemostasis or anastomosis	122
Venous grafting or bypass for the intra-abdominal veins	89
Venous thrombectomy (laparotomy)	66
Total	39,011

**Table 28:** Other vascular systems (total; 31,110 cases)

Procedure	Case
Peripheral arteriovenous fistula creation	22,180
Arterial grafting or bypass (other arteries)	4796
Peripheral arteriovenous fistula creation using synthetic graft	1616
Percutaneous angioplasty for an arteriovenous fistula (with stent graft)	1497
Arterial grafting or bypass for the head and neck arteries	594
Peripheral arteriovenous fistula creation with ulnar vein transposition	349
Vessel ligation (laparotomy)	231
Arterial grafting or bypass for the aorta	172
Lymphatic vessel anastomosis	139
Arterial grafting or bypass for the intrathoracic arteries	106
Vessel ligation (thoracotomy)	86
Percutaneous angioplasty for artery injury of an extremity (with stent graft)	78
Aortic repair or anastomosis	71
Percutaneous embolization (retroperitoneum) for emergency hemostasis	48
Venous injury repair or ligation of the intra-abdominal veins (excluding IVC)	37
Lymphaticovenous anastomosis	33
IVC injury repair with suture hemostasis	28
Percutaneous embolization for limb artery injury (emergency hemostasis)	25
Percutaneous embolization for intestinal or mesenteric bleeding (emergency hemostasis)	24
Total	32,110

*IVC* Inferior vena cava

**Table 29:** Other peripheral vascular surgeries (total; 44,036 cases)

Procedure	Case
Percutaneous venoplasty for limb veins	6258
Endovascular embolization for lower extremity varicose veins	6111
Arterial embolization	3378
Vascular exposure surgery (artery)	3109
Surgical removal of PCPS/ECMO	2780
Sclerotherapy for lower extremity varicose veins	2728
Vascular embolization (head, thoracic, or abdominal vessels)	2609
Venous graft harvesting (great saphenous vein)	2269
Venous graft harvesting	2253
Vessel ligation (non-thoracotomy or non-laparotomy)	2168
High ligation of lower extremity veins	2057
Internal thoracic artery harvesting	1271
Toe amputation	900
Arterial graft harvesting	896
Surgical insertion of PCPS/ECMO	772
Transfemoral amputation or above-knee amputation	552
Arteriovenous fistula takedown	516
Venoplasty (other veins)	449
Below-knee amputation	386
Percutaneous cardiopulmonary support (PCPS) insertion	367
Foot amputation	193
Venous anastomosis (other veins)	168
Percutaneous removal of intravascular foreign body	167
Surgical removal of intra-aortic balloon pump (IABP)	165
Radial artery harvesting	161
Portal vein embolization (via ileal vein)	158
Arterial reconstruction or anastomosis (intrathoracic artery, excluding aorta)	128
Surgical insertion of intra-aortic balloon pump (IABP)	127
Vascular foreign body removal via arteriotomy	109
Percutaneous embolization (pelvis)	100
Iliac artery aneurysm surgery	100
Inferior vena cava (IVC) filter removal	82
Percutaneous angioplasty for mesenteric vessels (with stent placement)	76
Autologous free composite tissue transplantation	73
Gastroepiploic artery harvesting	71
Arterial flap surgery	67
Free flap transplantation	51
Venoplasty and anastomosis for the finger veins (hand or foot)	50
Aortic coarctation surgery with Damus-Kaye-Stansel (DKS) anastomosis	49
Intra-aortic balloon pumping (every 3 h)	45
Portal vein embolization (transhepatic)	27
Thrombectomy (thoracotomy)	20
Portal vein branch ligation	20
Total	44,036

*PCPS* Percutaneous Cardiopulmonary Support, *ECMO* Extracorporeal Membrane Oxygenation, *IABP* intra-aortic balloon pump, *IVC* inferior vena cava

**Fig. 5:**
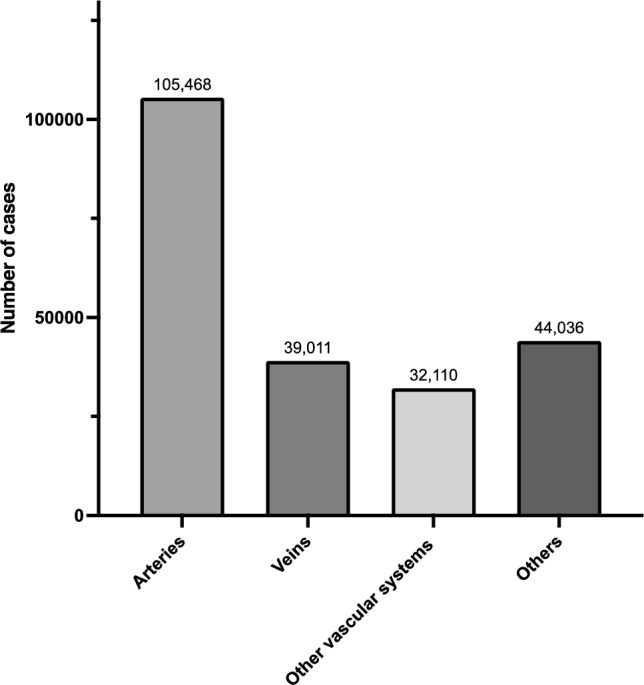
The number of cases in the category of peripheral vascular surgery

### (VI) Surgery of the head and neck/body surface/endocrine system

In 2023, 160,244 surgical procedures in the category of surgery of the head and neck/body surface/endocrine system were registered in the NCD. The procedure with the highest number in this category was wound treatment (≥ 6 years), length < 5 cm, not involving muscle or organ (*n* = 35,522), followed by skin incision (length < 10 cm) (*n* = 13,570), and incision and drainage of a perianal abscess (*n* = 9097).

Tables 30, 31, 32, 33 and Fig. 6 show the number of surgeries in each subcategory (skin and soft tissues; neck; thyroid and parathyroid glands; and adrenal and gonadal glands, respectively).

**Table 30:** Skin and soft tissues (total; 122,546 cases)

Procedure	Case
Wound treatment (≥ 6 years), length < 5 cm, not involving muscle or organ	35,522
Skin incision (length < 10 cm)	13,570
Incision and drainage of a perianal abscess	9097
Wound treatment (≥ 6 years, length 5–10 cm, not involving muscle or organ)	6595
Wound treatment (≥ 6 years, length < 5 cm, involving muscle or organ)	6132
Lymph node excision (diameter < 3 cm)	5600
Excision of a subcutaneous benign tumor (non-exposed area, diameter < 3 cm)	3488
Wound treatment (≥ 6 years, length 5–10 cm, involving muscle or organ)	3228
Excision of a benign skin tumor (non-exposed area, diameter < 3 cm)	3015
Lymph node excision (diameter ≥ 3 cm)	2783
Wound treatment (≥ 6 years, length ≥ 10 cm, involving muscle or organ)	2519
Excision of a subcutaneous benign tumor (non-exposed area, diameter 3–6 cm)	2356
Excision of a benign skin tumor (exposed area, diameter < 2 cm)	2271
Wound treatment (< 6 years, length < 2.5 cm, not involving muscle or organ)	1991
Simple ingrown nail surgery	1805
Excision of a benign skin tumor (non-exposed area, diameter 3–6 cm)	1714
Wound treatment (≥ 6 years, length ≥ 10 cm, not involving muscle or an organ)	1667
Excision of a subcutaneous benign tumor (exposed area, diameter < 2 cm)	1504
Excision of a subcutaneous benign tumor (exposed area, diameter 2–4 cm)	1345
Excision of a subcutaneous foreign body	1295
Incision and drainage of perirectal abscess	1065
Excision of a benign skin tumor (exposed area, diameter 2–4 cm)	1054
Excision of a subcutaneous benign tumor (non-exposed area, diameter ≥ 6 cm)	1028
Nail plate removal	979
Simple excision of a malignant skin tumor	910
Wound treatment (< 6 years, length 2.5–5 cm, not involving muscle or organ)	671
Ingrown nail surgery with nail bed/matrix involvement	529
Wound treatment (< 6 years, length 2.5–5 cm, involving muscle or organ)	505
Skin incision (length 10–20 cm)	494
Abdominal wall tumor excision (without reconstructive surgery)	489
Excision of a subcutaneous benign tumor (exposed area, diameter ≥ 4 cm)	447
Wound treatment (< 6 years, length 5–10 cm, involving muscle or organ)	418
Excision of a benign skin tumor (non-exposed area, diameter ≥ 6 cm)	416
Incision and drainage for felon (soft tissue infection)	413
Lymph node biopsy (needle puncture)	403
Excision of a subcutaneous hematoma	379
Wound treatment (< 6 years, length < 2.5 cm, involving muscle or organ)	364
Excision of a benign skin tumor (exposed area, diameter ≥ 4 cm)	360
Excision of a foreign body from muscle	262
Incision and drainage of abdominal wall abscess	256
Wound treatment (< 6 years, length ≥ 10 cm, involving muscle or organ)	249
Split-thickness skin graft (≥ 200 cm^2^)	249
Surgery for axillary osmidrosis (flap method)	247
Flap creation, transfer, resection, or delayed flap procedure (non-exposed, non-mucosal, non-joint area, < 25 cm^2^)	210
Skin biopsy	145
Full-thickness skin graft (< 25 cm^2^)	143
Full-thickness skin graft (25–100 cm^2^)	128
Split-thickness skin graft (100–200 cm^2^)	127
Excision of a benign soft tissue tumor (trunk)	122
Skin shaving (< 25 cm^2^)	118
Incisional biopsy of subcutaneous soft tissue tumor (trunk)	116
Excision of a breast foreign body	108
Skin incision (length ≥ 20 cm)	105
Split-thickness skin graft (25–100 cm^2^)	98
Open liver cyst fenestration	92
Stump revision (soft tissue only, toe)	82
Flap creation, transfer, resection, or delayed flap procedure (exposed area, < 25 cm^2^)	80
Autologous free composite tissue transplantation	73
Wound treatment (< 6 years, length 5–10 cm, not involving muscle or organ)	68
Arterial flap surgery	67
Scar contracture release	67
Pedicled musculocutaneous flap transfer	62
Foreign body removal from palm	56
Split-thickness skin graft (< 25 cm^2^)	51
Free flap transplantation	51
Incision and drainage of chest wall abscess	49
Flap creation, transfer, resection, or delayed flap procedure (exposed area, 25–100 cm^2^)	47
Foreign body removal from sole	47
Flap creation, transfer, resection, or delayed flap procedure (non-exposed, non-mucosal, non-joint area, ≥ 100 cm^2^)	45
Flap creation, transfer, resection, or delayed flap procedure (non-exposed, non-mucosal, non-joint area, 25–100 cm^2^)	40
Full-thickness skin graft (100–200 cm^2^)	39
Lymph node abscess incision and drainage	38
Excision of a superficial hemangioma (exposed area other than face or head, diameter < 3 cm)	34
Wound treatment (age under 6, length ≥ 10 cm, not involving muscle or organ)	33
Excision of a superficial hemangioma (non-exposed area, diameter < 3 cm)	29
Stump revision (soft tissue only, finger)	28
Wide excision of a malignant skin tumor	28
Simple resection of a malignant neck tumor	26
Incision and drainage of deep cervical abscess	26
Flap creation, transfer, resection, or delayed flap procedure (joint area, < 25 cm^2^)	25
Pedicled muscle flap transfer	24
Incisional biopsy of a soft tissue tumor (trunk)	24
Full-thickness skin graft (≥ 200 cm^2^)	24
Esophageal foreign body removal (cervical approach)	24
Mediastinal lymph node dissection (parasternal)	23
Flap creation, transfer, resection, or delayed flap procedure (joint area, ≥ 100 cm^2^)	20
Excision of benign soft tissue tumor (thigh)	20
Total	122,546

**Table 31:** Neck (total; 18,467 cases)

Procedure	Case
Tracheostomy	7264
Thyroidectomy (without lateral neck dissection)	2998
Axillary lymph node dissection	2954
Total or subtotal thyroidectomy (without lateral neck dissection)	1637
Total or subtotal thyroidectomy (with unilateral lateral neck dissection)	947
Thyroidectomy (with lateral neck dissection)	521
Cervical lymph node dissection	478
Unilateral neck dissection	376
Retroperitoneal lymph node dissection	245
Total or subtotal thyroidectomy (with bilateral lateral neck dissection)	236
Inguinal and femoral lymph node dissection	223
Bilateral neck dissection	178
Endoscopic thyroid resection for malignant thyroid tumor	161
Thyroglossal duct cyst excision	116
Excision of branchial fistula or branchial cyst	69
Endoscopic total or subtotal thyroidectomy for malignant thyroid tumor	37
Parotid tumor excision	27
Total	18,467

**Table 32:** Thyroid and parathyroid glands (total; 9802 cases)

Procedure	Case
Partial thyroidectomy (thyroid nodule excision, unilateral)	4186
Parathyroidectomy	1921
Total thyroidectomy for Graves' hyperthyroidism	1541
Partial thyroidectomy (thyroid nodule excision, bilateral)	718
Endoscopic hemithyroidectomy	409
Subtotal thyroidectomy for Graves' hyperthyroidism	258
Completion total thyroidectomy	186
Parathyroidectomy with autotransplantation	117
Fasciotomy or fascial incision	99
Endoscopic-assisted thyroidectomy	65
Thyroid biopsy via open incision	63
Endoscopic total thyroidectomy for Graves' disease	56
Endoscopic parathyroid tumor resection	46
Endoscopic bilateral thyroidectomy	45
Thyroid tissue biopsy via excisional biopsy	37
Laryngopharyngeal surgery for malignant tumor (with cervical, thoracic, or abdominal reconstruction)	31
Extensive parathyroid cancer resection	24
Total	9802

**Table 33:** Adrenal and gonadal glands (total; 9429 cases)

Procedure	Case
Orchiopexy for undescended testis (extra-abdominal)	4294
Circumcision (circular excision)	580
Repair of communicating hydrocele	551
Surgical repair of hydrocele of the canal of Nuck	550
Resection of a retroperitoneal malignant tumor	494
Orchiectomy	283
Laparoscopic resection of a retroperitoneal tumor	260
Extensive resection of a retroperitoneal malignant tumor	250
Ovarian tumor excision (open surgery)	236
Ovarian tumor excision (laparoscopic)	229
Surgery for testicular torsion (with contralateral orchiopexy)	199
Orchiopexy for undescended testis (intra-abdominal)	150
Circumcision (dorsal slit method)	134
Laparoscopic adrenal tumor resection	133
Laparoscopic orchiopexy for intra-abdominal undescended testis	128
Laparoscopic partial oophorectomy	106
Adrenalectomy	103
Surgery for testicular torsion (without contralateral orchiopexy)	101
Laparoscopic retroperitoneal tumor biopsy	89
Surgery for testicular torsion	78
Open partial oophorectomy	74
Orchiopexy for contralateral testis during testicular torsion	61
Circumcision (frenuloplasty or phimosis repair)	57
Open adrenal tumor resection	55
Orchiectomy for undescended testis	41
Laparoscopic resection of an adrenal malignant tumor	40
Simple adrenal malignant tumor resection (including pheochromocytoma)	36
Adrenal tumor resection (medullary tumor, pheochromocytoma)	34
Laparoscopic orchiectomy for undescended testis	31
Vasectomy or vas resection	27
Extensive resection of adrenal malignant tumor	25
Total	9429

**Fig. 6:**
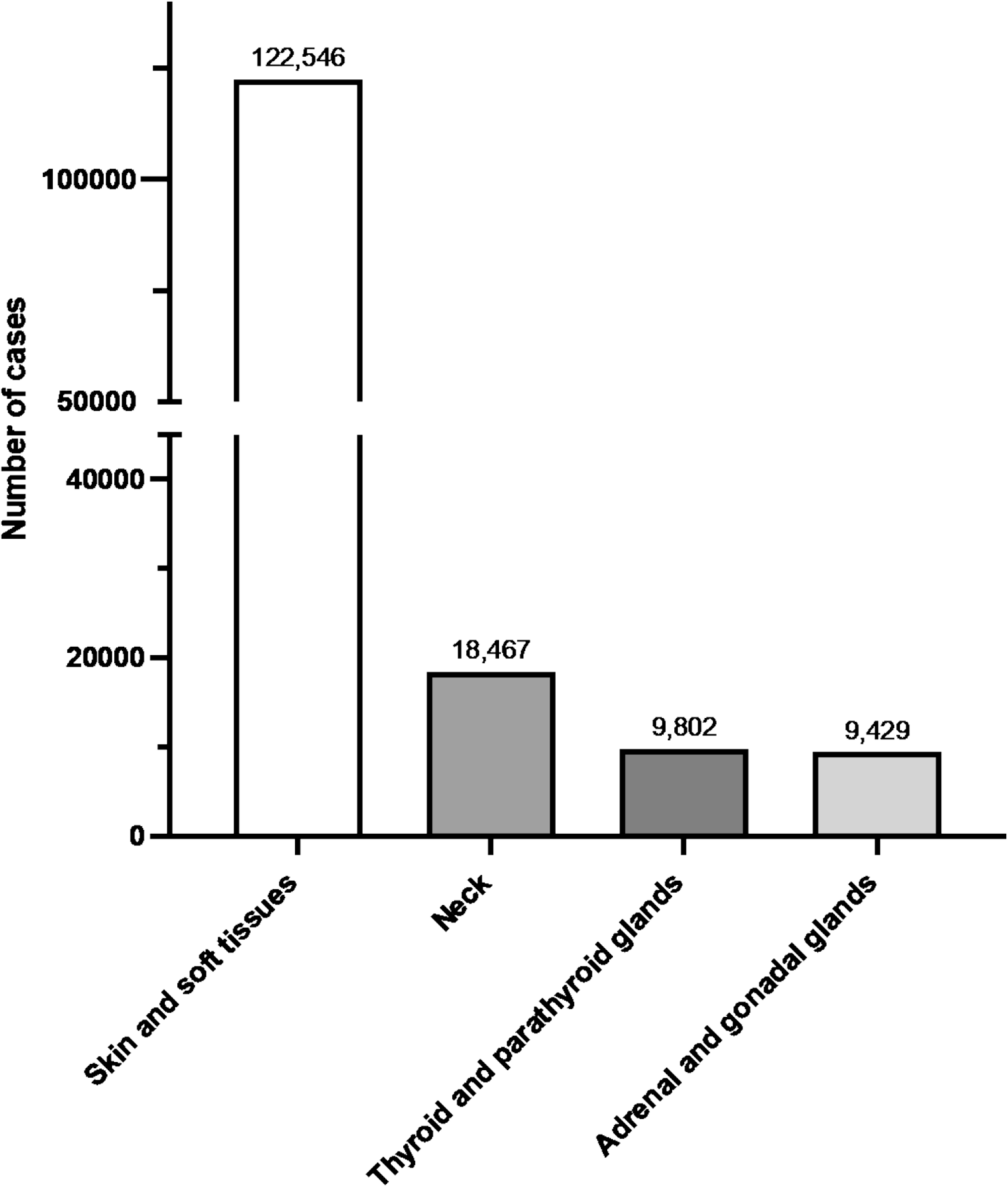
The number of cases in the category of surgery of the head and neck/body surface/endocrine system

### (VII) Pediatric surgery

In 2023, 43,164 pediatric surgical procedures were registered in the NCD. The procedure with the highest number in this category was laparoscopic inguinal hernia surgery (*n* = 8599), followed by open inguinal hernia surgery (*n* = 5240), and orchiopexy for undescended testis (extra-abdominal) (*n* = 4276). Tables 34, 35, 36, 37, 38, 39, 40, 41 and Fig. 7 show the number of surgeries in each subcategory (body surface; thorax; diaphragm; gastroenterological system; nutritional management; tumor; transplantation; and other pediatric surgical procedures, respectively).

**Table 34:** Body surface (total; 24,038 cases)

Procedure	Case
Laparoscopic inguinal hernia surgery	8599
Open inguinal hernia surgery	5240
Orchiopexy for undescended testis (extra-abdominal)	4276
Umbilical hernia surgery (rectus abdominis diastasis)	3751
Circumcision (circular excision)	511
Surgery for hydrocele of the canal of Nuck	154
Excision of a benign skin tumor (exposed area, diameter < 2 cm)	150
Orchiopexy for intra-abdominal testis	147
Excision of a subcutaneous benign tumor (exposed area, diameter < 2 cm)	137
Excision of a subcutaneous benign tumor (non-exposed area, diameter < 3 cm)	132
Laparoscopic orchiopexy for intra-abdominal testis	128
Circumcision (dorsal slit)	126
Excision of a benign skin tumor (non-exposed area, diameter < 3 cm)	113
Excision of a subcutaneous benign tumor (exposed area, diameter 2–4 cm)	103
Abdominal wall reconstruction (abdominal wall rupture or omphalocele)	97
Excision of a benign skin tumor (exposed area, diameter 2–4 cm)	61
Open abdominal wall incisional hernia surgery	59
Excision of branchial fistula or cyst	52
Open orchiectomy for undescended testis	36
Excision of a subcutaneous benign tumor (non-exposed area, diameter 3–6 cm)	35
First-stage omphalocele repair	33
Laparoscopic orchiectomy for undescended testis	27
Excision of subcutaneous benign tumor (non-exposed area, diameter ≥ 6 cm)	24
Excision of lymphangioma (diameter ≥ 3 cm)	24
Excision of a benign skin tumor (non-exposed area, diameter 3–6 cm)	23
Total	24,038

**Table 35:** Thorax (total; 824 cases)

Procedure	Case
Endoscopic pectus excavatum repair	155
Pectus excavatum repair (sternal elevation)	110
Pectus bar removal	104
Exploratory thoracotomy	101
Thoracoscopic lobectomy	84
Thoracoscopic wedge resection (two or more sites) for pulmonary cyst	65
Open lobectomy	62
Thoracoscopic mediastinal tumor resection	47
Open mediastinal tumor resection	35
Thoracoscopic debridement for empyema	34
Thoracoscopic lung plication	27
Total	824

**Table 36:** Diaphragm (total; 136 cases)

Procedure	Case
Congenital diaphragmatic hernia surgery (transabdominal, direct suture)	55
Congenital diaphragmatic hernia surgery (transabdominal, with synthetic patch)	43
Thoracoscopic surgery for congenital diaphragmatic hernia (direct suture)	38
Total	136

**Table 37:** Gastroenterological system (total; 12,000 cases)

Procedure	Case
Laparoscopic simple appendectomy	4164
Laparoscopic complex appendectomy	1281
Laparoscopic-assisted gastrostomy (including percutaneous endoscopic, percutaneous, and open approaches)	527
Stoma creation	519
Exploratory laparotomy for diagnosis and biopsy	338
Stoma closure with bowel resection	313
Open gastrostomy	310
Open small bowel resection	305
Incision and drainage of perianal abscess	300
Open appendectomy	290
Pyloromyotomy for hypertrophic pyloric stenosis	251
Low perineal anorectal reconstruction for imperforate anus	239
Acute diffuse peritonitis surgery with drainage of intraperitoneal abscess	229
Open surgery for intestinal adhesions	201
Surgery for intestinal malrotation	183
Radical surgery for complex fistula-in-ano	164
Enterostomy creation	147
Endoscopic gastrostomy creation	146
Laparoscopic Hirschsprung disease surgery	130
Definitive surgery for esophageal atresia (Gross type C)	109
Surgery for duodenal atresia or stenosis (diamond anastomosis)	108
Meckel’s diverticulectomy	96
Laparoscopic surgery for intestinal adhesions	95
Surgery for biliary atresia	91
Surgery for congenital intestinal atresia (with bowel resection)	87
Open reduction for intussusception	87
Surgery for choledochal cyst	86
Laparoscopic small bowel resection	82
Open pyloroplasty	76
Enterostomy closure with bowel resection	69
Laparoscopic surgery for choledochal cyst	64
Laparoscopic reduction for intussusception	60
Placement of abdominal drain	60
Hirschsprung disease surgery	57
Open limited colectomy	56
Laparoscopic pyloromyotomy for hypertrophic pyloric stenosis	54
Localized intraperitoneal abscess surgery (appendiceal abscess)	54
Stoma closure without bowel resection	45
Laparoscopic splenectomy	43
Laparoscopic high anorectal reconstruction for imperforate anus	42
Laparoscopic limited colectomy	39
Intermediate anorectal reconstruction for imperforate anus (sacroperineal approach)	38
Anal polyp resection	37
Laparoscopic hiatal hernia surgery	37
Laparoscopic surgery for intestinal malrotation	37
Robot-assisted surgery for choledochal cyst	31
Esophageal variceal ligation	29
Surgery for intestinal duplication	29
Surgery for congenital intestinal atresia (without bowel resection)	27
Percutaneous gastrostomy creation	26
Open gastric perforation repair1	25
Intermediate anorectal reconstruction for imperforate anus (PSARP)	24
Thoracoscopic esophageal atresia repair	23
Surgery for duodenal atresia or stenosis (non-diamond anastomosis)	20
Laparoscopic surgery for gastric volvulus	20
Total	12,000

**Table 38:** Nutritional management (total; 2131 cases)

Procedure	Case
Placement of central venous nutrition port (head, neck, or other sites)	1781
Central venous nutrition catheter placement (venotomy)	251
Placement of central venous nutrition port (limbs)	99
Total	2131

**Table 39:** Tumors (total; 561 cases)

Procedure	Case
Ovarian tumor excision (open surgery)	95
Ovarian tumor excision (laparoscopic surgery)	66
Adnexal tumor excision (open surgery)	50
Excision of omental, mesenteric, and retroperitoneal tumors (without bowel resection)	48
Laparoscopic partial oophorectomy	48
Adnexal tumor excision (laparoscopic surgery)	47
Simple nephrectomy for renal malignancy	34
Partial liver resection	32
Laparoscopic detorsion of ovarian torsion	28
Sacrococcygeal teratoma surgery (superficial)	25
Sacrococcygeal teratoma surgery (pelvic cavity)	25
Simple orchiectomy for testicular malignancy (including high inguinal orchiectomy)	22
Renal biopsy (excisional)	21
Laparoscopic resection of adrenal malignant tumor	20
Total	561

**Table 40:** Transplantation (total; 198 cases)

Procedure	Case
Living donor partial liver transplantation	102
Allogeneic kidney transplantation	40
Back table surgery for liver transplantation (living donor)	28
Liver procurement from brain-dead donor	28
Total	198

**Table 41:** Other pediatric surgeries (total; 3276 cases)

Procedure	Case
Lingual frenuloplasty	475
Esophageal dilation (balloon catheter)	388
Hypospadias repair	332
Vesicoureteral reflux surgery (collagen and hyaluronic acid injection)	275
Ureteral stent removal	194
Excision of urachal remnant	194
Vesicoureteral reflux surgery	179
Excision of accessory auricle	111
Labial frenuloplasty	107
Peritoneal dialysis catheter placement	105
Sclerotherapy for superficial lymphangioma	100
Laryngotracheal separation	96
Excision of congenital auricular sinus	84
Ureteral reimplantation with anti-reflux mechanism	82
Pyeloplasty (including ureteropelvic junction repair)	77
Ureteral stent placement	75
Laparoscopic pyeloplasty (including ureteropelvic junction repair)	57
Surgery for buried penis	55
Sclerotherapy for deep lymphangioma	53
Labial adhesion repair (excluding simple adhesion release)	44
Thoracic diaphragm suture (without patch reconstruction)	39
Cystostomy creation	38
Surgery for laryngeal stenosis (T-tube insertion)	31
Nephrostomy creation	29
Open nephrectomy	28
Ureteral stent exchange	28
Total	3276

**Fig. 7:**
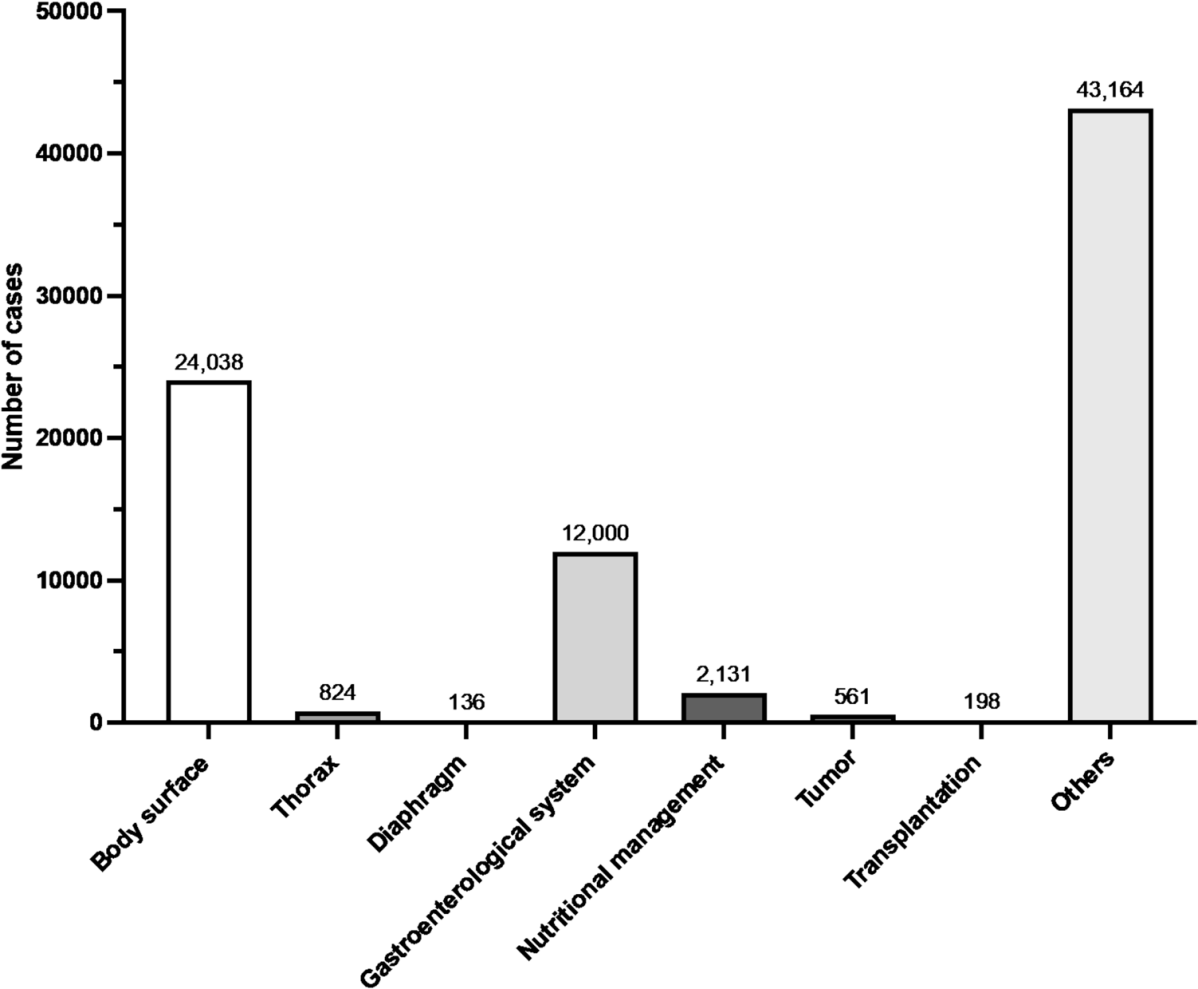
The number of cases in the category of pediatric surgery
